# Supplementary material for: Single-cell profiling reveals pervasive heterogeneity in subcellular RNA localization
Source: bioRxiv. 2026 Feb 6:2026.02.04.703776. Preprint. [Version 1] doi: 10.64898/2026.02.04.703776 (PMC13119314; doi:10.64898/2026.02.04.703776)
Supplement: 1 [file NIHPP2026.02.04.703776v1-supplement-1.pdf]

Figure S1

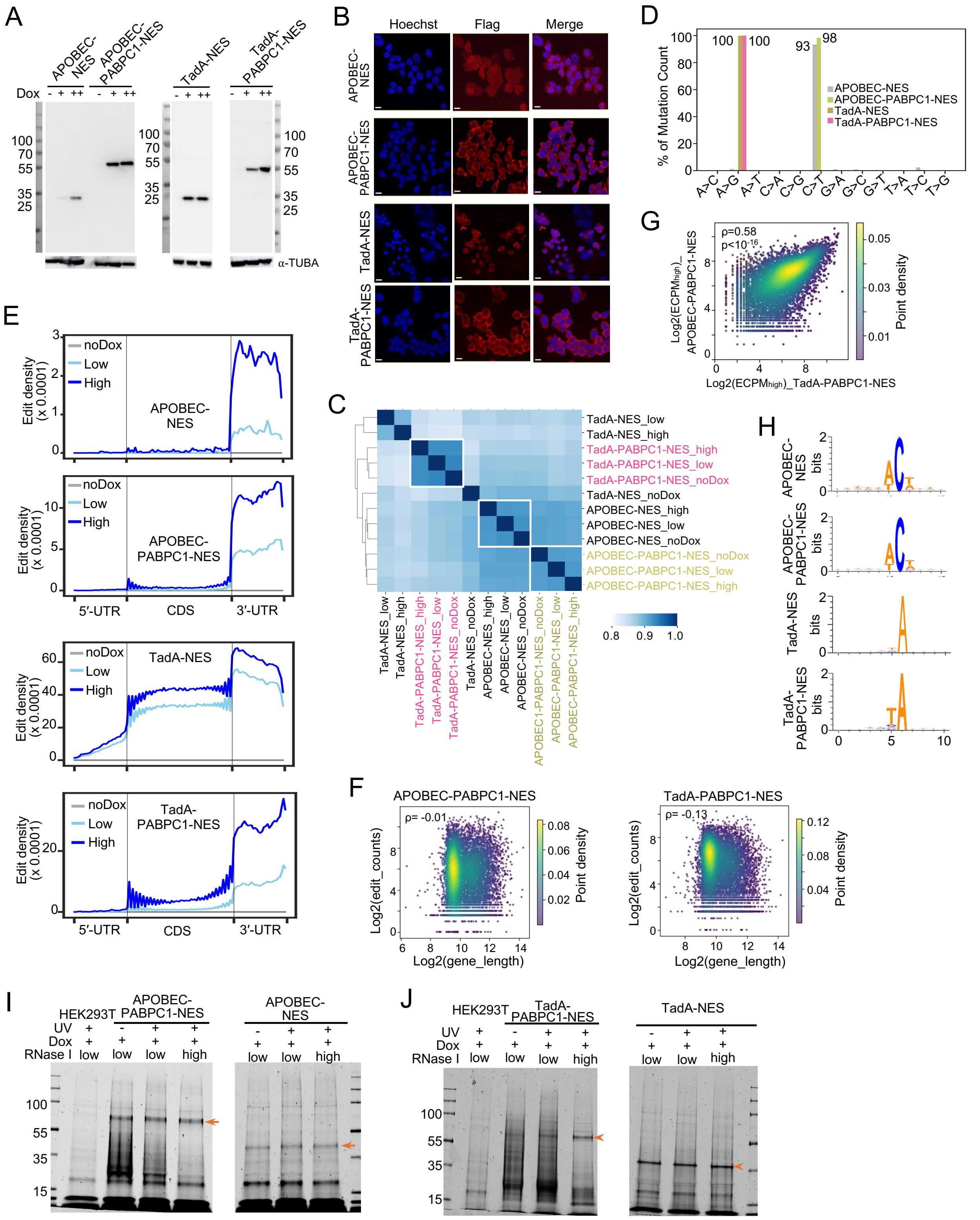

A
APOBEC-NES
APOBEC-PABPC1-NES
TadA-NES
TadA-PABPC1-NES
Dox - + ++ - + ++ - + ++ - + ++
100
70
55
35
25
α-TUBA
B
Hoechst
Flag
Merge
APOBEC-NES
APOBEC-PABPC1-NES
TadA-NES
TadA-PABPC1-NES
C
TadA-NES_low
TadA-NES_high
TadA-PABPC1-NES_high
TadA-PABPC1-NES_low
TadA-PABPC1-NES_noDox
TadA-NES_noDox
APOBEC-NES_high
APOBEC-NES_low
APOBEC-NES_noDox
APOBEC-PABPC1-NES_noDox
APOBEC-PABPC1-NES_low
APOBEC-PABPC1-NES_high
APOBEC1-PABPC1-NES_noDox
0.8
0.9
1.0
D
% of Mutation Count
100
100
93
98
APOBEC-NES
APOBEC-PABPC1-NES
TadA-NES
TadA-PABPC1-NES
A>C
A>G
A>T
C>A
C>G
C>T
G>A
G>C
G>T
T>A
T>C
T>G
E
Edit density (x 0.0001)
noDox
Low
High
APOBEC-NES
APOBEC-PABPC1-NES
TadA-NES
TadA-PABPC1-NES
5′-UTR
CDS
3′-UTR
F
APOBEC-PABPC1-NES
TadA-PABPC1-NES
ρ= -0.01
ρ= -0.13
Log2(edit_counts)
Log2(gene_length)
Point density
G
ρ=0.58
p<10-16
Log2(ECPMhigh)_APOBEC-PABPC1-NES
Log2(ECPMhigh)_TadA-PABPC1-NES
Point density
H
APOBEC-NES
APOBEC-PABPC1-NES
TadA-NES
TadA-PABPC1-NES
bits
I
HEK293T
APOBEC-PABPC1-NES
APOBEC-NES
UV
Dox
RNase I
low
high
J
HEK293T
TadA-PABPC1-NES
TadA-NES
UV
Dox
RNase I
low
high

Figure S1. Characterization of RLP reporters in the cytoplasm. **A**, Western blot of APOBEC1-PABPC1-NES, TadA-PABPC1-NES, and control reporters lacking RBD of PABPC1 (APOBEC1-NES, TadA-NES) in stable cell lines 24 h after doxycycline induction, detected with anti-FLAG antibody. Cells were treated with no (−), low (+), or high (++) doxycycline. α-TUBA served as a loading control. Molecular weights (kDa) are indicated. **B**, Immunofluorescence (IF) images of the same reporters in panel **A**, collected 24 h after doxycycline induction. Nuclei were stained with Hoechst (blue) and reporter expression detected by FLAG immunostaining (red). Scale bars: 15 μm (APOBEC1-NES, APOBEC1-PABPC1-NES, and TadA-PABPC1-NES); 30 μm (TadA-NES). Fewer cells were observed in TadA-NES. **C**, Pairwise Pearson correlation heatmap of transcriptome profiles ($\log_{10}$ TPM) for each reporter under no, low, or high doxycycline induction. **D**, Single-nucleotide variant (SNV) distributions across 12 substitution types in cells expressing each reporter. **E**, Metagene plots of normalized editing density across the 5′ UTR, coding sequence (CDS), and 3′ UTR. Regions are scaled to relative length on the x-axis; normalized edit density is shown on the y-axis. Profiles are shown under no, low, and high doxycycline conditions. **F**, Scatter plots of $\log_2$ (gene length) *vs.* $\log_2$(edit counts) in cells expressing APOBEC1-PABPC1-NES (left) or TadA-PABPC1-NES (right). Spearman correlation coefficients (ρ) are indicated. **G**, Scatter plot of gene-level editing abundance (ECPM) between TadA-PABPC1-NES and APOBEC1-PABPC1-NES reporters under high doxycycline induction. Spearman correlation coefficients (ρ) and p value are indicated. **H**, Sequence logos showing nucleotide contexts surrounding editing sites for each reporter. **I-J**, Fluorescence imaging of PAR-CLIP gels for APOBEC1-PABPC1-NES and control APOBEC1-NES (panel **I**), or TadA-PABPC1-NES and control TadA-NES (panel **J**). Samples were prepared under no UV, low (0.015 U/μl), or high (0.15 U/μl) RNase I digestion. Untransfected controls were included. Orange arrows mark excised bands used for RNA-seq library preparation; corresponding gel regions from untransfected controls were processed in parallel.

Figure S2

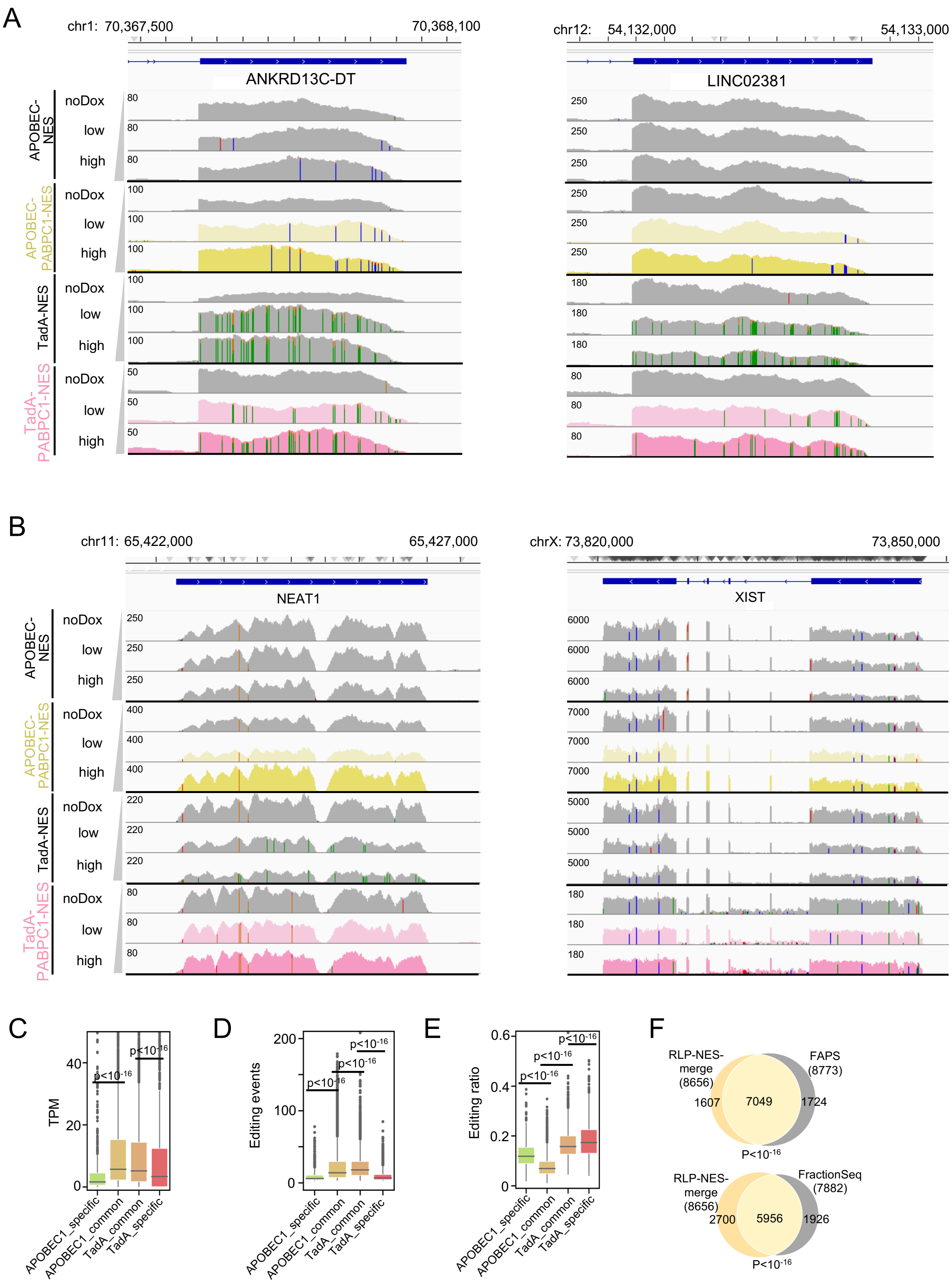
A
chr1: 70,367,500
70,368,100
ANKRD13C-DT
chr12: 54,132,000
54,133,000
LINC02381
APOBEC-NES
APOBEC-PABPC1-NES
TadA-NES
TadA-PABPC1-NES
noDox
low
high
B
chr11: 65,422,000
65,427,000
NEAT1
chrX: 73,820,000
73,850,000
XIST
C
TPM
p<10-16
APOBEC1_specific
APOBEC1_common
TadA_common
TadA_specific
D
Editing events
E
Editing ratio
F
RLP-NES-merge (8656)
FAPS (8773)
1607
7049
1724
P<10-16
FractionSeq (7882)
2700
5956
1926

Figure S2. Characterization of cytoplasmic RNAs detected by RLP reporters. A-B, The editing profiles (RNA-seq coverage track) showing representative lncRNAs in cytoplasmic RLP datasets. Panel **A**, Representative edited lncRNAs ANKRD13C-DT and LINC02381, panel **B**, Representative non-edited lncRNAs NEAT1 and XIST. Read coverages are shown for APOBEC1-PABPC1-NES (yellow), TadA-PABPC1-NES (pink), and control (gray) reporters under no, low, or high doxycycline induction. Colored bars in coverage tracks indicate editing events (C-to-U for rAPOBEC1, A-to-G for TadA; SNV detection threshold = 0.05). Nucleotide colors: A = green, C = blue, G = orange, T = red (reversed on minus strand). **C-E**, Box plots comparing RNAs detected only by APOBEC1-PABPC1-NES (APOBEC1_specific), only by TadA-PABPC1-NES (TadA_specific), or by both reporters (APOBEC1_common, TadA_common). “APOBEC1_common” and “TadA_common” indicate shared RNAs, with features taken from the APOBEC1-PABPC1-NES and TadA-PABPC1-NES datasets, respectively. Panel **C**, Transcript abundance (TPM). Panel **D**, Number of editing events. Panel **E**, Editing ratio. P values are indicated. **F**, Overlap of cytoplasmic RNAs detected by both RLP reporters with those identified in published cytosolic fractionation datasets. P values are indicated.

Figure S3

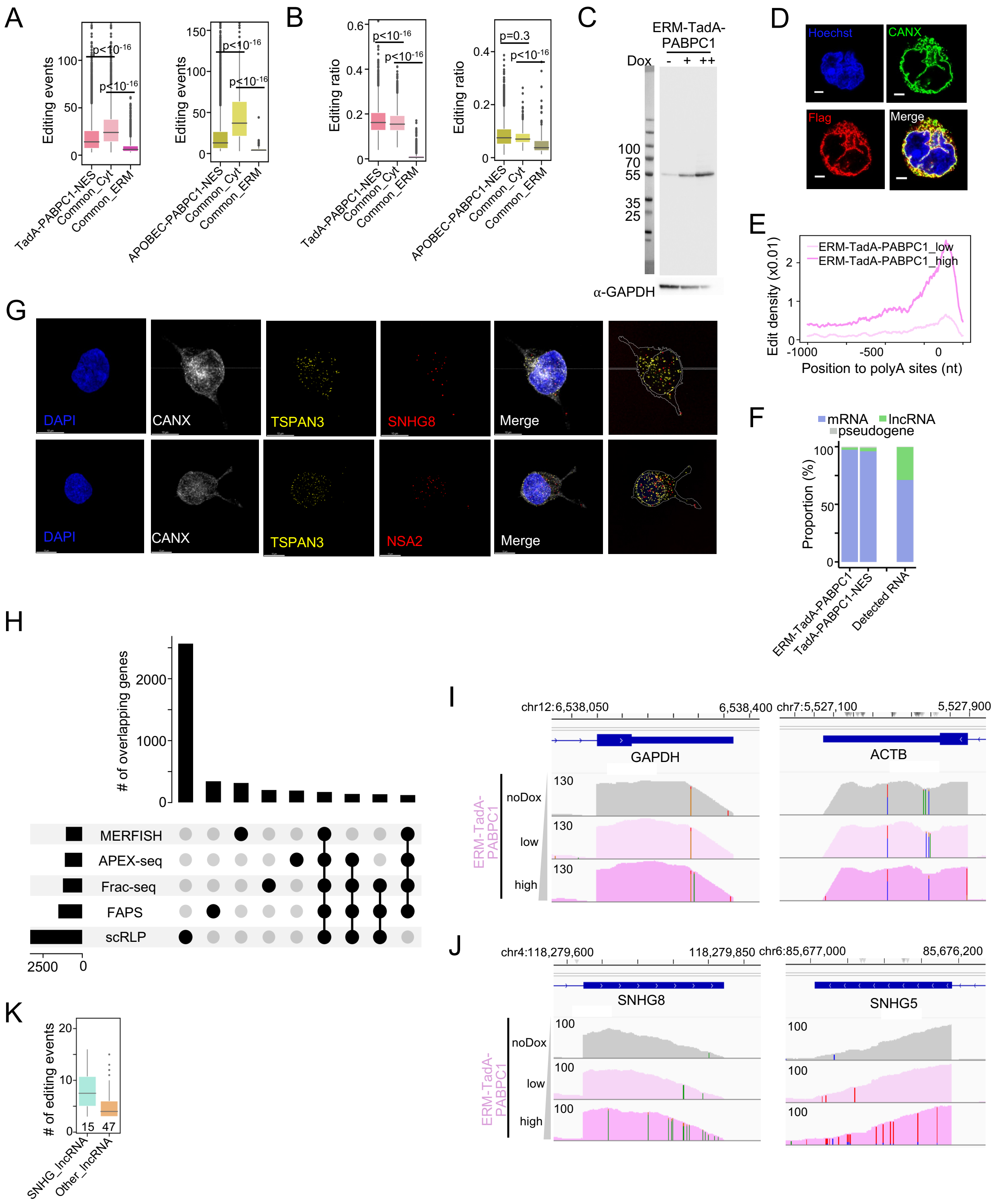

Figure S3. Characterization of the RLP reporter in the endoplasmic reticulum membrane (ERM). A-B, Distribution of (panel **A**) editing events and (panel **B**) editing ratios for three RNA groups: RNAs detected only by TadA-PABPC1-NES or APOBEC1-PABPC1-NES in the

cytoplasm, RNAs shared between cytoplasm and ERM with values from TadA-PABPC1-NES or APOBEC1-PABPC1-NES (Common_Cyt), and RNAs shared between cytoplasm and ERM with values from ERM-TadA-PABPC1 or ERM-APOBEC1-PABPC1 (Common_ERM). P values are indicated. **C**, Western blot of ERM-TadA-PABPC1 expression in stable cell line 24 h after doxycycline induction, detected with anti-FLAG antibody. Cells were treated with no (−), low (+), or high (++) doxycycline. α-GAPDH served as a loading control; Molecular weights (kDa) are indicated on the left. **D**, IF images of ERM-TadA-PABPC1 reporter 24 h after induction. Nuclei stained with Hoechst (blue), ER with CANX (green), and reporter with FLAG immunostaining (red). Scale bars, 2 μm. **E**, Metagene analysis of editing density relative to poly(A) sites. **F**, Composition of detected ERM-associated RNAs compared with cytoplasm RNAs and total RNA-seq. Bars show the proportions of edited mRNAs (blue), lncRNAs (green), and pseudogenes (gray) in ERM-TadA-PABPC1, TadA-PABPC1-NES, and total detected RNAs by RNA-seq. **G**, Representative multiplex FISH-IF validation of ERM-associated RNAs from z-stack images. DAPI (blue), CANX (gray), ERM control RNA TSPAN3 (yellow), and test ERM RNAs (red; SNHG8 and NSA2). Rightmost panels show nuclear and ER masks (gray) overlaid with RNA puncta (yellow/red). Scale bar, 10 μm. See **Supplementary Videos 4–5**. **H**, Overlap of ERM-associated transcripts identified by RLP, FAPS, APEX-seq, Fractionation-seq (Frac-seq), and MERFISH. The top nine intersections by gene count are shown. **I-J**, The editing profiles (RNA-seq coverage track) of panel **I**, GAPDH and ACTB and panel **J**, representative ERM-associated SNHG family RNAs SNHG8 and SNHG5, from ERM-TadA-PABPC1 reporter under no, low, or high doxycycline induction. Colored bars indicate editing events (SNV detection threshold = 0.05). **K**, The number of editing events detected in SNHG family lncRNAs compared with other ERM-associated lncRNAs.

Figure S4

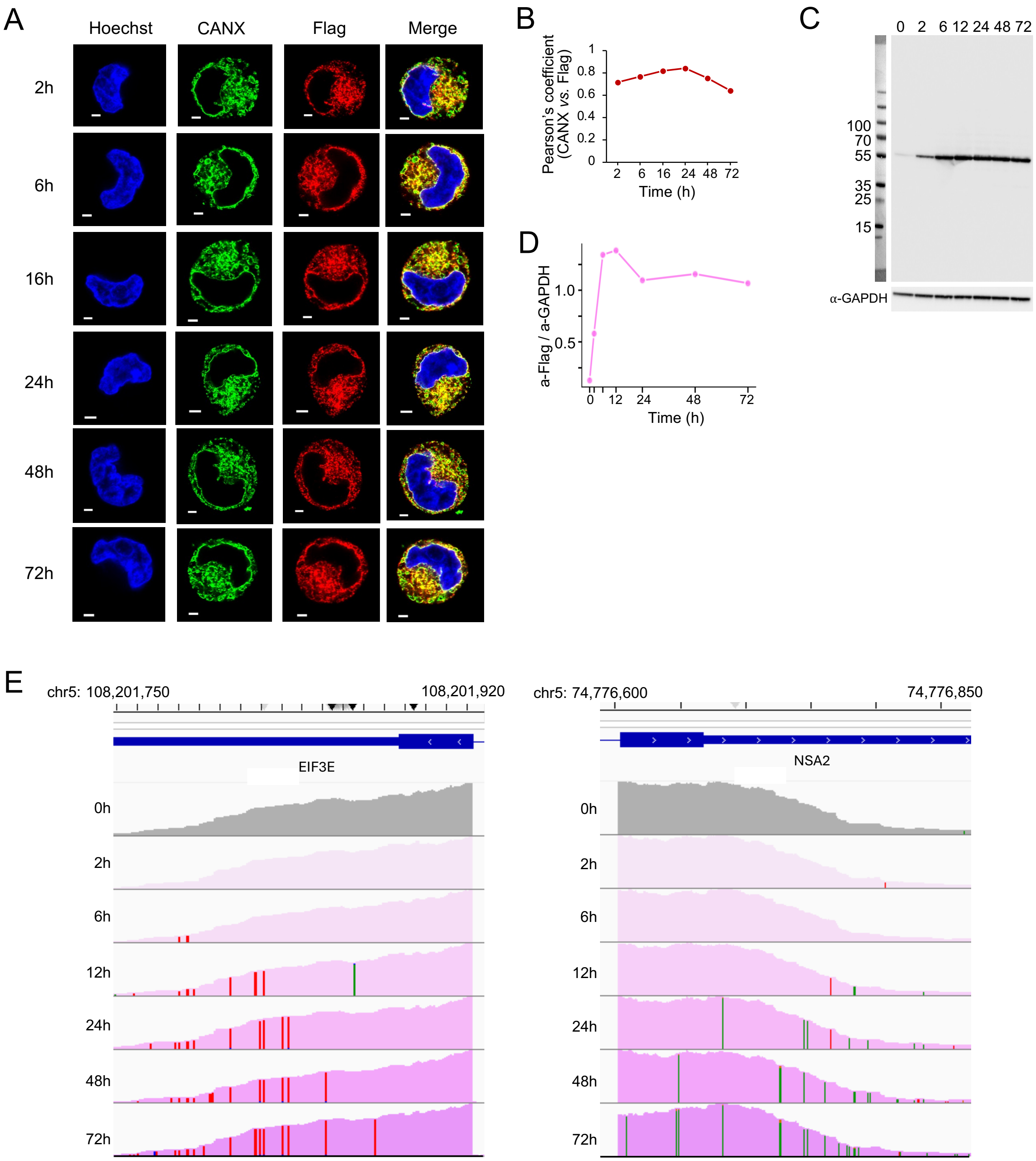

Figure S4. Time-course analysis of the RLP reporters at the ERM. **A**, IF images of ERM-TadA-PABPC1 from 2-72 h after doxycycline induction. Nuclei were stained with Hoechst (blue), ER with CANX (green), and reporter with FLAG immunostaining (red). Scale bars, 2 μm. **B**, Pearson's correlation coefficients between CANX (green) and FLAG (red) signals in 3D Z-stacks acquired on the Leica TCS SP8. N ⩾ 8 cells per time point. **C**, Western blot of ERM-TadA-PABPC1 expression from 0-72h after induction (0 h, uninduced control), detected with anti-FLAG

antibody. α-GAPDH served as a loading control; molecular weights (kDa) are indicated. **D**, Quantification of FLAG signal normalized to GAPDH (α-FLAG/α-GAPDH) at the indicated times, corresponding to panel **C**. **E**, The editing profiles (RNA-seq coverage track) of representative ERM-associated cluster 2 RNAs (EIF3E and NSA2). Colored bars indicate editing events (A-to-G for TadA; SNV detection threshold = 0.02).

Figure S5

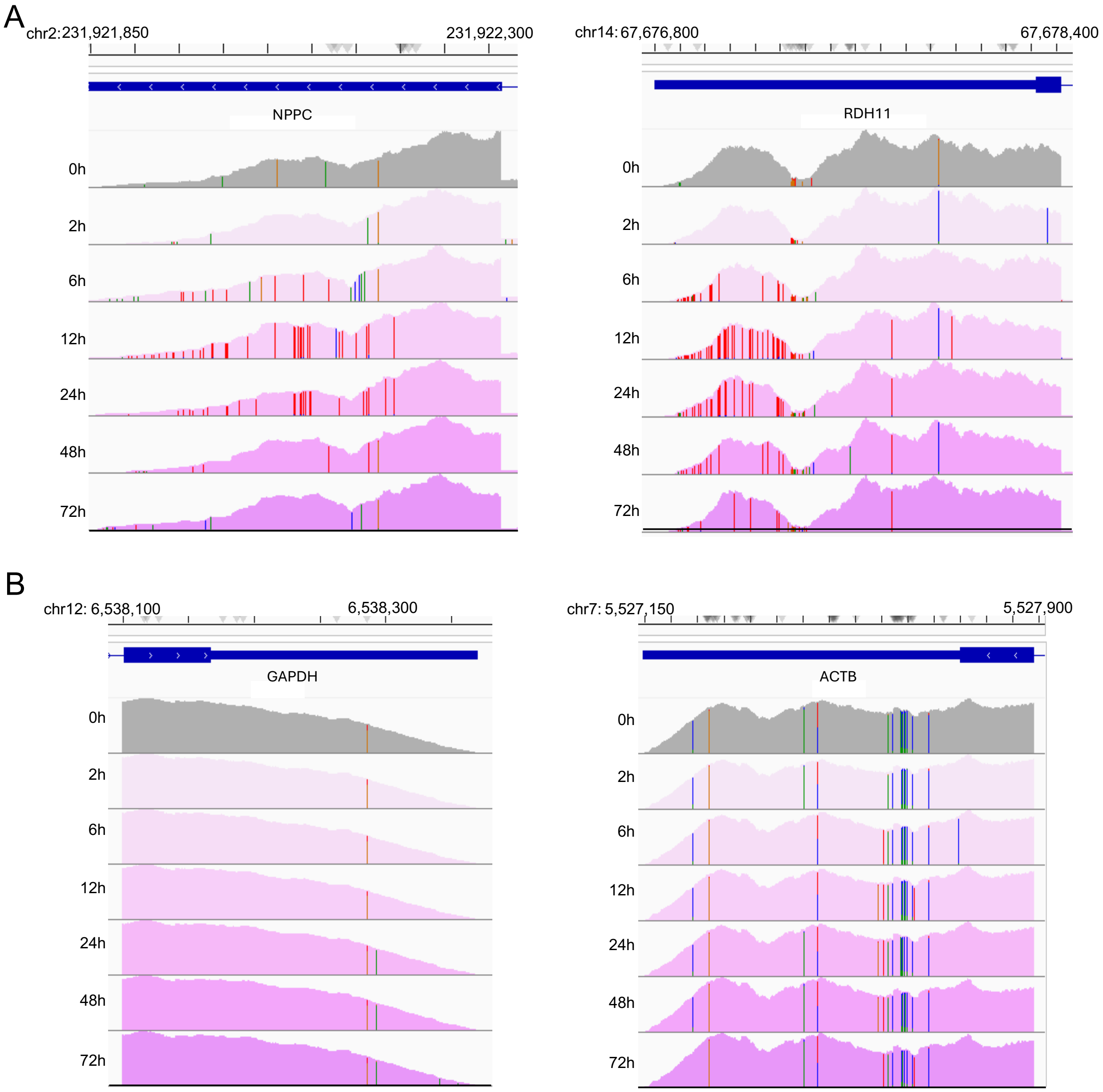

Figure S5. The editing profiles of representative RNAs. A, ERM-associated cluster 4 RNAs NPPC and RDH11. **B**, Cytoplasmic RNAs GAPDH and ACTB. For both panels, read coverages are shown for ERM-TadA-PABPC1 at 0 h (gray) and 2–72 h (purple). Colored bars indicate editing events (A-to-G for TadA; SNV detection threshold = 0.02).

Figure S6

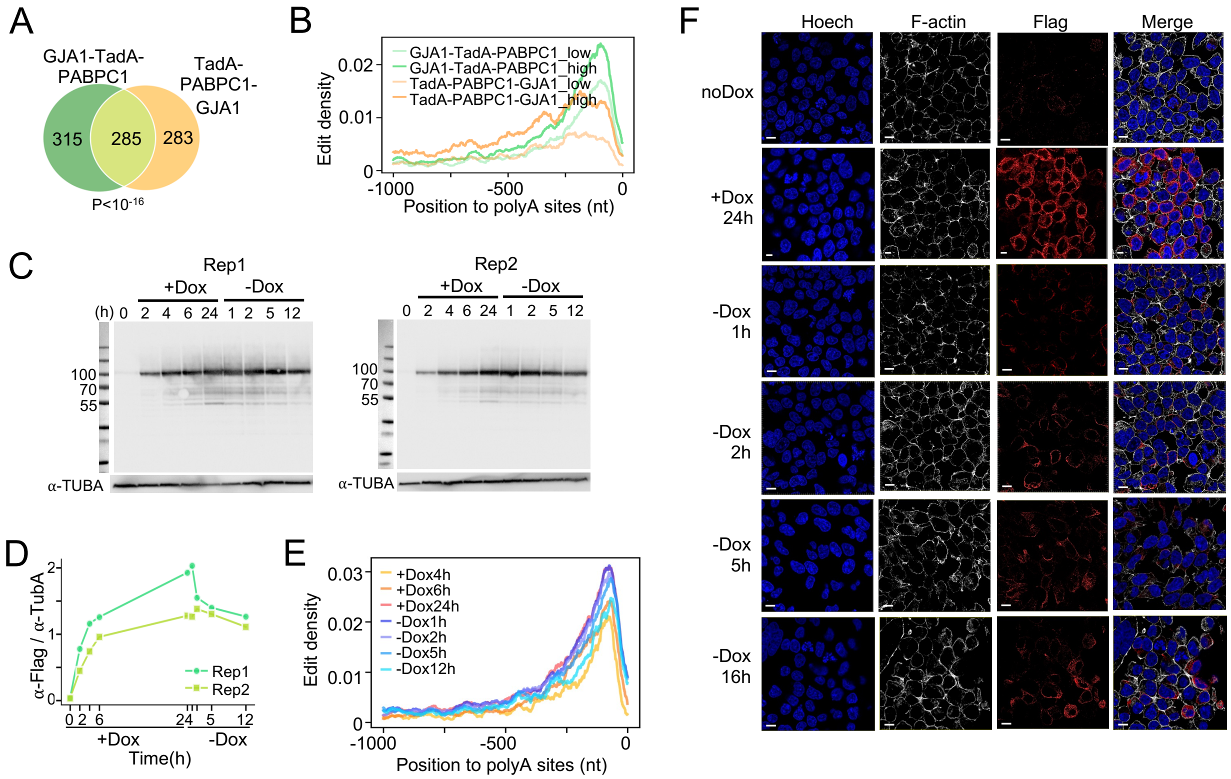

Figure S6. Characterization of RLP reporter at the plasma membrane (PM). A, Overlap of RNAs identified by GJA1-TadA-PABPC1 and TadA-PABPC1-GJA1 reporters. P values are indicated. **B**, Metagene profiles of editing density relative to poly(A) sites for GJA1-TadA-PABPC1 and TadA-PABPC1-GJA1 reporters. **C**, Western blot of GJA1-TadA-PABPC1 reporter across the dual-phase experiment in two biological replicates. The reporter was detected with anti-FLAG antibody. α-TUBA served as a loading control; molecular weights (kDa) are indicated. **D**, Quantification of reporter expression by FLAG signal normalized to α-TUBA (α-FLAG/α-TUBA) at the indicated times, corresponding to panel **C**. **E**, Metagene profiles of editing density relative to poly(A) sites during the dual-phase experiment. **F**, IF images of GJA1-TadA-PABPC1 reporter in the dual-phase experiment. Nuclei are stained with Hoechst (blue), F-actin with phalloidin (gray), and reporter with FLAG immunostaining (red). A high-resolution single-cell image acquired 1 h after doxycycline removal is shown in **Supplementary Video 6.** Scale bars, 10 μm.

Figure S7

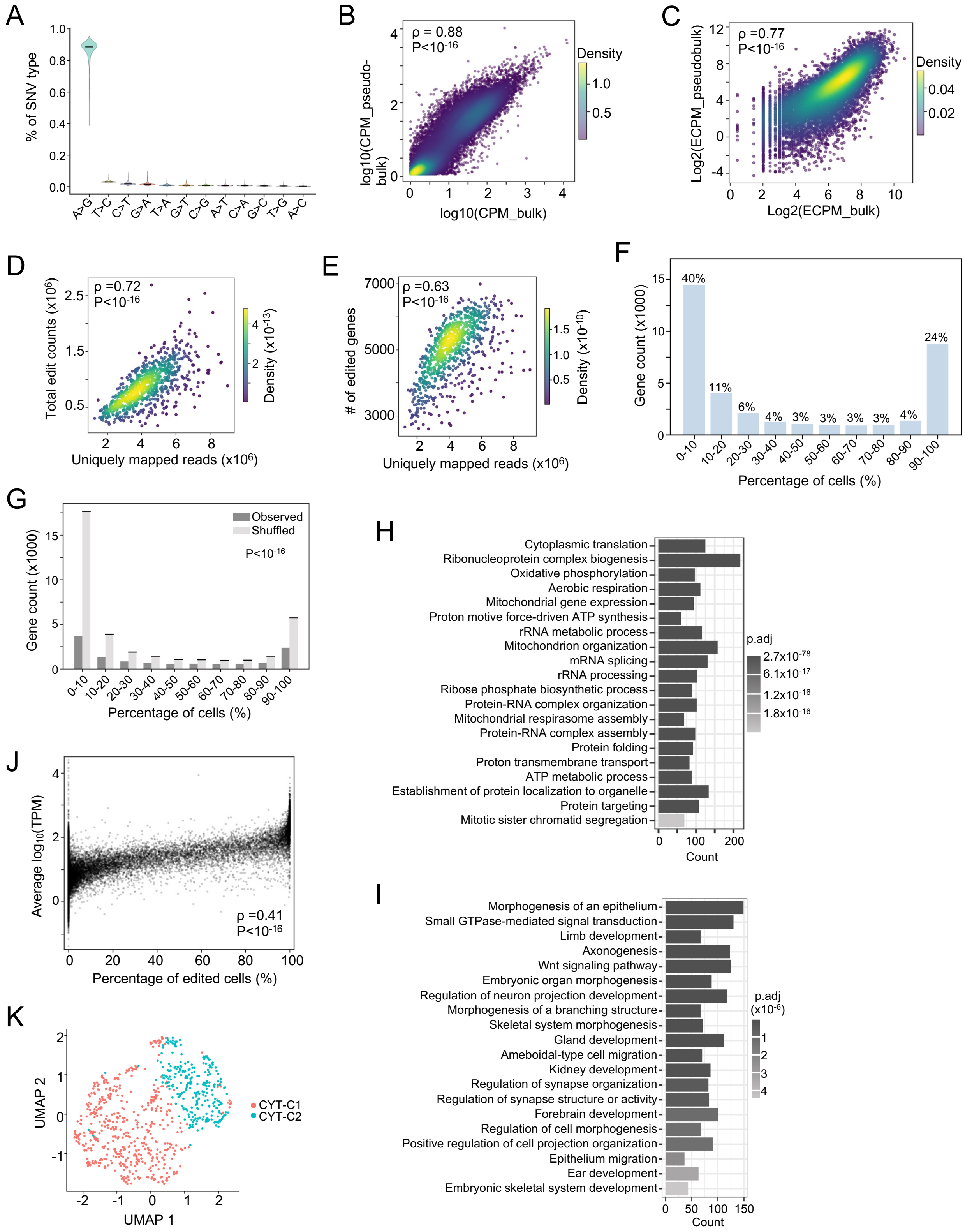

Figure S7. Characterization of cytoplasmic RNAs detected by scRLP-seq. A, SNV distributions across 12 substitution types among single cells expressing TadA-PABPC1-NES. **B**,

Correlation of expression between bulk and pseudobulk scRLP-seq ($\log_{10}$ CPM). Spearman ρ and p value are indicated. **C**, Correlation of editing level between bulk and pseudobulk scRLP-seq data ($\log_2$ ECPM). Spearman ρ and p value are indicated. **D-E**, Scatter plots of uniquely mapped reads *vs.* edit counts (panel **D**) or edited genes (panel **E**) per cell. **F**, Distribution of all detected genes by scRLP-seq (y-axis) binned by detection frequency across cells (x-axis). Percentages relative to the total number of detected genes are shown above. **G**, Genes were binned by detection rate and compared with three independent shuffling-based controls. Bars show observed data (gray) and shuffled controls (light gray, mean ± SD). **H-I**, GO Biological Process enrichment of RNAs detected in >90% (panel **H**) or <10% (panel **I**) of cells. **J**, Relationship between RNA abundance and the detection frequency across cells. **K**, UMAP of single cells based on editing profiles.

Figure S8

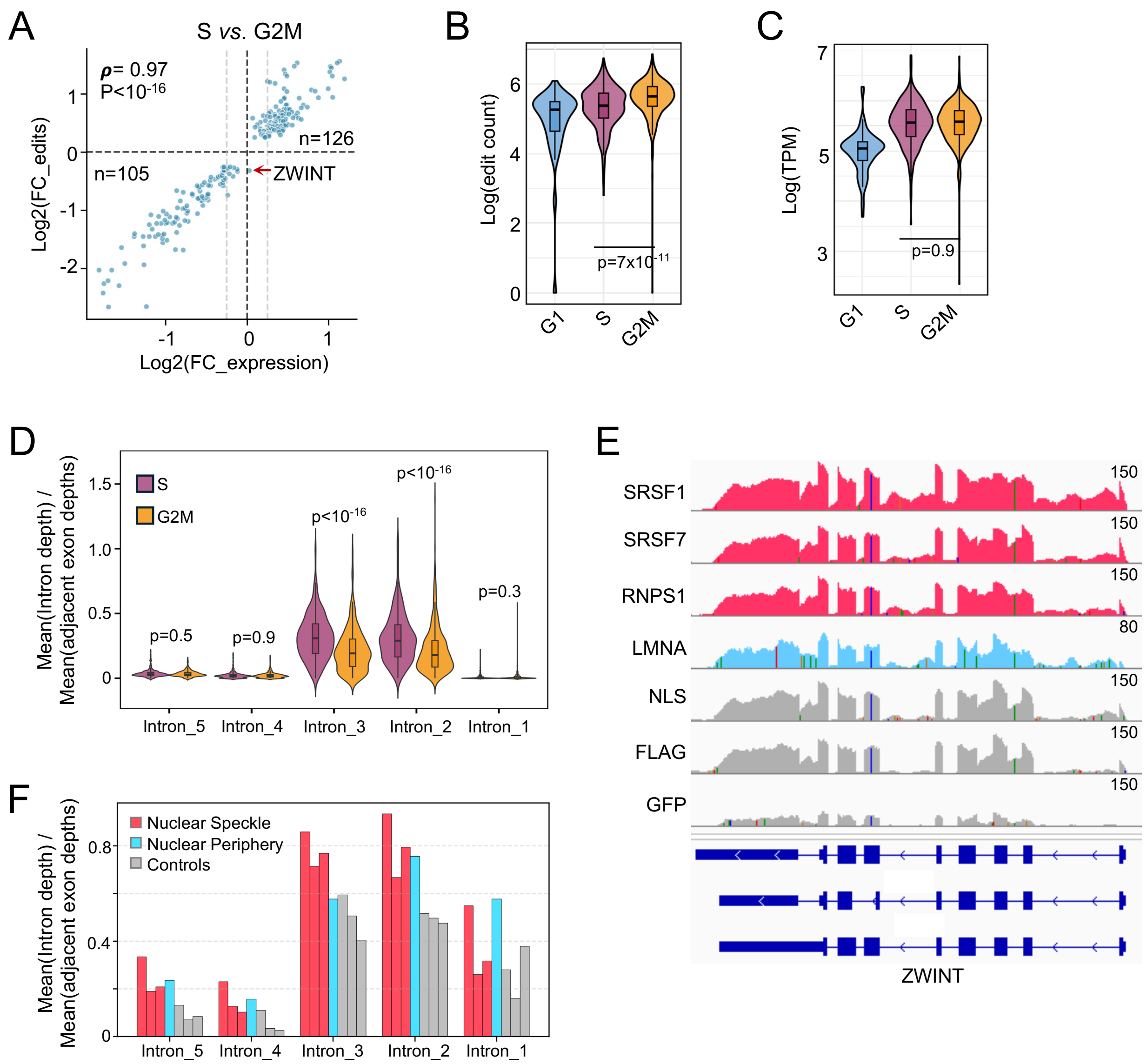

Figure S8. Redistribution of ZWINT mRNA during the S–G2/M transition. A, Scatter plot of $\log_2$ fold change (FC) of expression (x-axis) *vs.* editing (y-axis) for genes differing between S and G2/M phases (adjusted $p < 0.05$; $|\log_2 FC| \geqslant 0.25$). Dashed lines indicate zero and ±0.25 thresholds. Spearman ρ and p value are shown. Quadrant counts indicate gene numbers. ZWINT is highlighted (red arrow). **B-C**, The distributions of ZWINT editing (panel **B**) and expression (panel **C**) across single cells in G1, S, and G2/M phases. **D**, Distribution of intron ratios for constitutive introns of ZWINT across single cells measured by scRLP-seq. **E**, Genome tracks showing raw RNA-seq reads from the APEX2-nuclear marker and control samples mapped to the ZWINT locus. **F**, Intron ratio of ZWINT calculated from panel **E**. Nuclear speckle markers: SRSF1, SRSF7, and RNPS1; nuclear periphery marker: LMNA; controls: NLS, FLAG, and GFP.

Figure S9

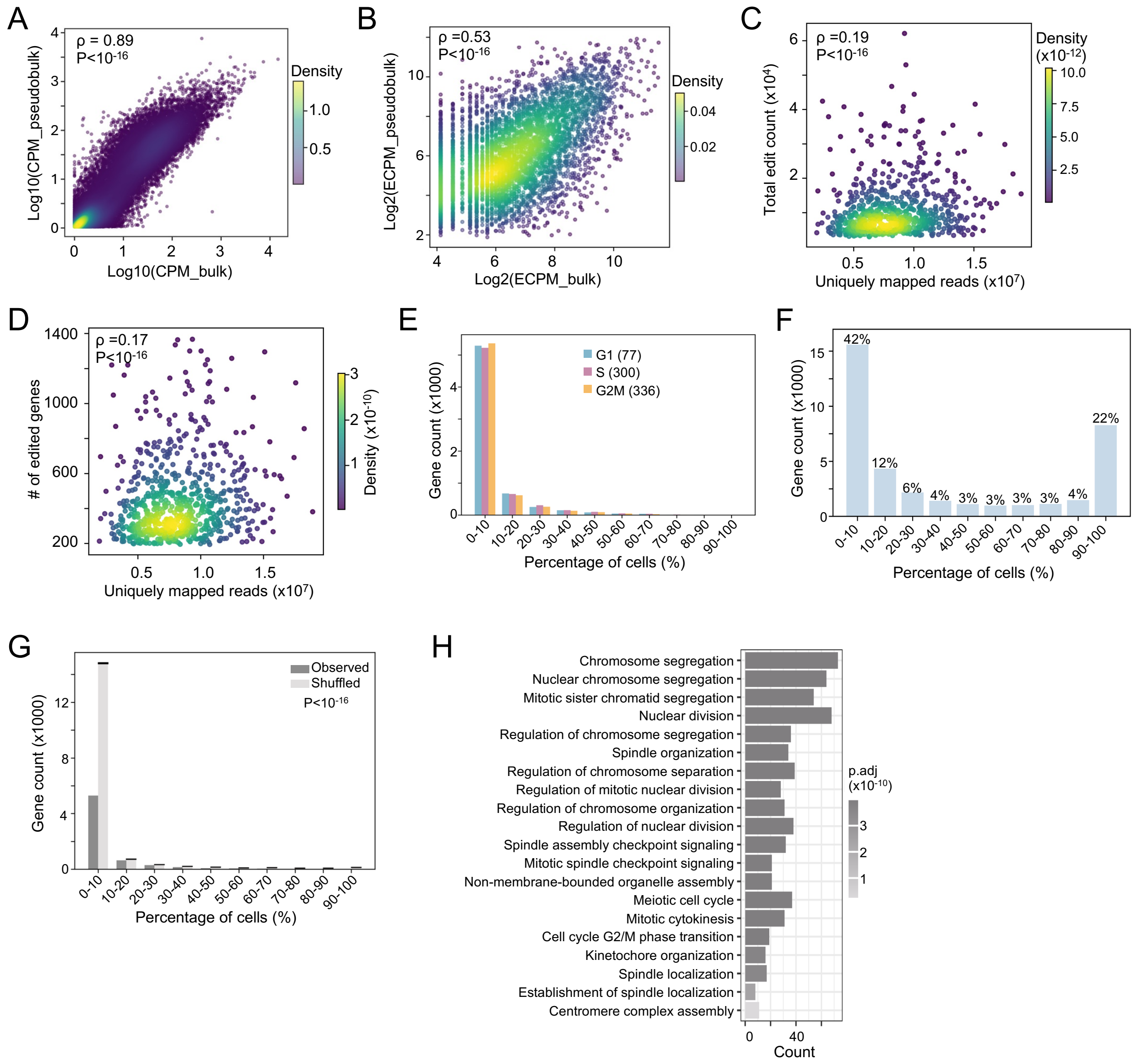

Figure S9. Characterization of ERM-associated RNAs detected by scRLP-seq. A-B, Correlation analysis of expression ($\log_{10}$ CPM, panel **A**) or editing ($\log_2$ ECPM, panel **B**) between bulk and pseudobulk scRLP-seq data. Spearman ρ and p value are indicated. **C-D**, Scatter plots of uniquely mapped reads *vs.* edit counts (panel **C**) or edited genes (panel **D**) per cell. **E**, Distribution of edited gene counts (y-axis) by detection frequency across cells (x-axis) for G1, S, and G2/M phases. **F**, Distribution of all genes detected by scRLP-seq (y-axis) binned by detection frequency across cells (x-axis). Percentages relative to the total number of detected genes are shown above. **G**, Genes were binned by detection rate (fraction of cells with $\geqslant 3$ editing events) in scRLP-seq data and compared with three independent shuffling simulations based on TPM-derived read distributions. Bars show the number of edited genes per bin for the observed data (gray) and the

shuffled controls (light gray, mean ± SD). **H**, GO Biological Process enrichment of the top 200 most variable genes from the expression-based UMAP in **Figure 7D**.
